# Prolonged Exposure to Neonatal Hyperoxia Impairs Neuronal and Oligodendrocyte Maturation Associated with Long-Lasting Neuroinflammatory Responses in Juvenile Mice

**DOI:** 10.3390/cells14151141

**Published:** 2025-07-24

**Authors:** Stefanie Obst, Meray Serdar, Karina Kempe, Dharmesh Hirani, Ursula Felderhoff-Müser, Josephine Herz, Miguel A. Alejandre Alcazar, Ivo Bendix

**Affiliations:** 1 Department of Paediatrics I, Neonatology and Experimental Perinatal Neurosciences, Centre for Translational Neuro- and Behavioural Sciences (C-TNBS), University Hospital Essen, University Duisburg-Essen, 45147 Essen, Germany; 2 Institute for Lung Health (ILH), Cardiopulmonary Institute (CPI), Member of the German Centre for Lung Research, University of Giessen and Marburg Lung Center, 35392 Giessen, Germany; 3 Translationale Experimental Pediatrics, Department of Pediatric and Adolescent Medicine, Faculty of Medicine, University of Cologne, 50937 Cologne, Germany; 4 Cologne Excellence Cluster for Stress Responses in Ageing-Associated Diseases (CECAD) and Center for Molecular Medicine Cologne (CMMC), Faculty of Medicine, University of Cologne, 50937 Cologne, Germany

**Keywords:** brain injury, neuroinflammation, hyperoxia, bronchopulmonary dysplasia, delayed neural maturation

## Abstract

Preterm infants often require oxygen supplementation, resulting in high risk for bronchopulmonary dysplasia (BPD) and neurodevelopmental deficits. Despite a growing number of studies, there is still little knowledge about brain injury in BPD models. Therefore, we exposed neonatal C57BL/6 mice to 85% oxygen from birth to postnatal day (P) 14. At P28, two weeks after recovery under normoxic conditions, right hemisphere was used for the analysis of mRNA and the left hemisphere for protein expression of neuronal cells, neuroinflammatory and vascularisation markers, analysed by real-time PCR and Western blot, respectively. Hyperoxia led to an altered expression of markers associated with neuronal and oligodendrocyte maturation and neuroinflammation such as *Dcx*, *Nestin*, *Il-1β*, *Il-6*, NG2, and YM1/2. These changes were accompanied by an increased expression of genes involved in angiogenesis and vascular remodelling, e.g., *Vegf-a*, *Nrp-1*, and *Icam-1.* Together, 14 days of hyperoxia triggered a phenotypic response, resembling signs of encephalopathy of prematurity (EoP).

## 1. Introduction

Premature birth affects approximately 10% of all pregnancies worldwide and represents a leading cause of neonatal mortality and morbidity [1]. Preterm infants are exposed to relative hyperoxia *ex utero* compared to *in utero* conditions, which can disrupt the development of immature organs such as the lung and the brain [2]. Common long-term consequences of prematurity include bronchopulmonary dysplasia (BPD) and encephalopathy of prematurity (EoP) and associated neurological deficits [3,4]. Hyperoxia-mediated brain and lung injury has been studied in experimental rodent models, although the protocols for mimicking injury differ significantly between these organs [5]. Neonatal brain injury can be induced by short-term hyperoxia (e.g., ~24 h with ~80% O_2_ starting around postnatal day (P) 6), whereas modelling mild or severe BPD typically requires prolonged hyperoxia exposure (up to 14 days with >60% O_2_) initiated around the time of birth [6,7]. Notably, children with BPD often exhibit adverse neurodevelopmental outcomes [8]. To date, investigations into brain injury in BPD models are sparse. However, a few recent studies were conducted immediately after hyperoxia exposure, representing developmental stages equivalent to newborns and toddlers. Though, these studies revealed disturbed maturation of neurons, oligodendrocytes, and altered vascularisation in the brain, the extent of impaired maturation of neural cells and the vasculature following the cessation of hyperoxia exposure remain unclear [9,10,11,12,13,14]. Information on neuroinflammatory responses is lacking, particularly in relation to whether markers of microglia activation, typically associated with signs of EoP, remain modulated in older animals. This study focussed on long-lasting effects of postnatal hyperoxia on developmental markers of neurons, oligodendrocytes, microglia, and vascularisation in a BPD model with 14 days of hyperoxia, followed by analyses at P28, equivalent to brain development of late childhood [15].

## 2. Materials and Methods

Animal studies were performed in accordance with the German regulations and legal requirements, approved by the local government authorities (LANUV, NRW, Germany; 81–02.04.2020.A095). C57BL/6 J mice were housed in humidity- and temperature-controlled rooms exposed to a 12 h dark/light cycle and were allowed food and water ad libitum.

### 2.1. Neonatal Hyperoxia-Induced Lung Injury Model

Since the severity of BPD and the concentrations of oxygen that preterm infants received are associated with an adverse neurocognitive outcome [16,17], we evaluated a neonatal hyperoxia-based mild-to-severe BPD model. For hyperoxia-induced lung injury, newborn mice were exposed to hyperoxia, as previously described [6]. In brief, newborn mice were grouped and randomly assigned by a blinded investigator to dams on the day of birth (born within 12 h of each other). Half of the litters were exposed to 85% O_2_ (hyperoxia, HO) in a hyper-/hypoxia chamber from Biospherix, with a ProOx 110 Cytocentric^®^ High Infusion Rate O_2_ Controller from Biospherix (Biospherix, Parish, NY, USA), while the other pups remained at room air (21% O_2_; normoxia, NO). Nursing dams were rotated daily between hyperoxia and room-air litters to avoid oxygen toxicity on the dams. Neonatal mice were exposed to hyperoxia or normoxia from birth to postnatal day 14 (P14) followed by recovery for another two weeks at normoxia. A total of 15 pups were used in this study (derived from two independent experiments; NO: *n* = 7 animals (2 females and 5 males); HO: *n* = 8 animals (5 females and 3 males)), no exclusion criteria were set *a priori*. At P28, mice were sacrificed, brains were excised and immediately snap-frozen and stored at −80 °C. Left and right hemispheres were used for isolation of protein and RNA, respectively. Experiments were performed by blinded investigators.

### 2.2. Immunoblotting

Protein isolation and blotting were performed as described previously [14]. Briefly, the left hemisphere was homogenised in ice-cooled radioimmunoprecipitation assay (RIPA) buffer (Sigma-Aldrich, St. Louis, MO, USA) containing protease inhibitor (Roche, Basel, Switzerland), followed by an incubation for 20 min on ice and centrifugation at 17,000× *g* for a further 20 min at 4 °C. Concentrations of the cytosolic supernatants were quantified using a BCA Kit (Thermo Fisher Scientific, Waltham, MA, USA). Per lane, 20 µg of protein was separated in 10% and 12.5% polyacrylamide gels and was transferred to a 0.2 µm nitrocellulose membrane (Amersham, Piscataway, NJ, USA). The equal loading and qualitative transfer of proteins were confirmed via the Ponceau S solution staining of the membranes. Non-specific antibody binding sites were blocked with 5% bovine serum albumin (BSA), 0.1% Tween 20 in Tris-buffered saline (TBS-T) for neural glial-antigen 2 (NG2), or in 5% non-fat milk power (Cell Signaling, Danvers, MA, USA) or TBS-T for all other antibodies. Membranes were incubated with primary antibodies at 4 °C overnight followed by incubation with the appropriate peroxidase-conjugated secondary antibodies in TBS-T (Table S1). Antibody binding was detected with enhanced chemiluminescence (GE healthcare Life Science, München, Germany). Densitometric analysis was performed with ImageLab software (version 5.2.1, BioRad, Feldkirchen, Germany) and proteins of interest were normalised to the reference protein β-ACTIN. Full western blots are provided in Figure S1.

### 2.3. Real-Time PCR

Gene expression analysis was performed as described previously using Taqman Real-Time PCR Assays (Thermo Fisher Scientific, Dreieich, Germany) [18]. Briefly, the right hemisphere of the brain was used for RNA isolation with TRIzol/chloroform extraction using the QIAzol reagent and DNase I treatment (both from Qiagen, Hilden, Germany). In total, 4.8 µg of RNA was reverse transcribed using SuperScript II Reverse Transcriptase (Invitrogen, Carlsbad, CA, USA). PCR products were quantified using assay-on-demand primers and TaqMan^TM^ Fast Advanced Master Mix (Applied Biosystems/Thermo Fisher Scientific, Table S2). Target gene expression was normalised to the housekeeping gene beta-2-microglobulin (*B2m*) and calculated using the 2^−ΔΔCT^ formula [19]. Additionally, delta CT values are shown in Figure S2.

### 2.4. Statistical Analysis

Statistical analysis was performed with GraphPad Prism 9 (GraphPad Software, Boston, MA, USA). Gaussian distribution was tested using the D’Agostino and Pearson omnibus normality test and data were analysed either by unpaired Students *t*-test (parametric) or the Mann–Whitney test (non-parametric). Data are presented as box plots with individual data points including median values, as well as the 25% and the 75% percentile. In all analyses, *p*-values less than 0.05 were considered to be statistically significant.

## 3. Results

### 3.1. Hyperoxia Compromised Neuronal Maturation Accompanied by Vascular Response

**Figure 1:**
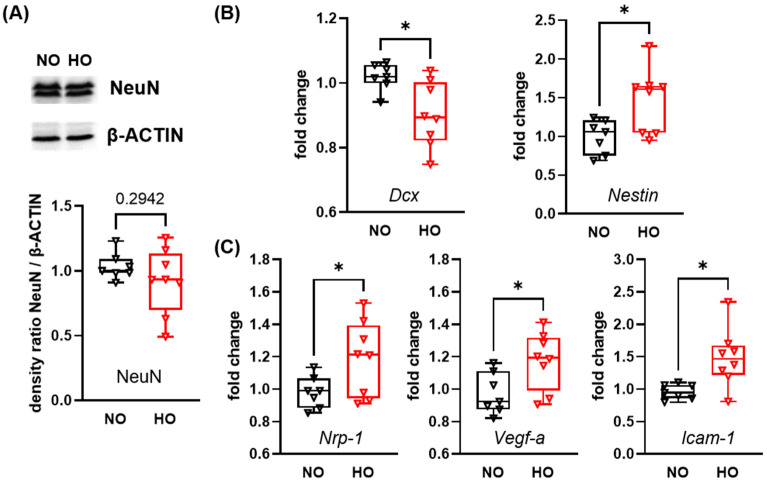
Alteration in expression levels of genes associated with neuronal maturation and vascularisation after 14 days of perinatal hyperoxia. Neonatal mice were exposed to 85% oxygen or room-air (21% oxygen) from P0 to P14 and remained under room-air conditions for 14 additional days. Brains were analysed via western blot for the postmitotic neuronal marker NeuN (**A**). mRNA expression analysis for genes involved in neuronal maturation (doublecortin (*Dcx*) and *Nestin*) (**B**) and vascular responses (neuropilin-1 (*Nrp-1*), vascular endothelial growth factor-a (*Vegf-a*), and intercellular adhesion molecule-1 (*Icam-1*)) (**C**) was performed via real-time PCR. Data are presented as box plots with individual data points including median values, as well as the 25% and the 75% percentile. NO: *n* = 7 animals; HO: *n* = 8 animals; * *p* < 0.05.

To analyse the impact of 14 days of hyperoxia on neuronal development at P28, we performed western blot analysis of the left hemisphere for the postmitotic marker NeuN, revealing no difference between the normoxia and hyperoxia group (Figure 1A). However, mRNA expression of doublecortin (*Dcx*), a marker for cortical neuronal precursor cells and immature neurons was reduced, while the expression of the intermediate filament protein *Nestin* on neuroepithelial stem cells was upregulated in hyperoxia-treated animals (Figure 1B). In addition, neither growth factors, important in the normal brain development such as epidermal growth factor (*Egf*), insulin-growth factor-1 (*Igf*) or fibroblast growth factor 2 (*Fgf2*) (Figure S3A) nor synaptic molecules like synaptophysin (*Syn*), calbindin-1 (*Calb*) and neuregulin-1 (*Nrg-1*) were altered after hyperoxia at P28 (Figure S3B). Interestingly, vascular response markers were increased, including neuropillin-1 (*Nrp-1*) involved in vascular sprouting, the angiogenesis factor *Vegf-a* and the intercellular adhesion molecule-1 (*Icam-1*) (Figure 1C).

### 3.2. Hyperoxia Leads to an Impaired Oligodendrocyte Maturation and a Long-Lasting Alteration of Neuroinflammatory Responses

**Figure 2:**
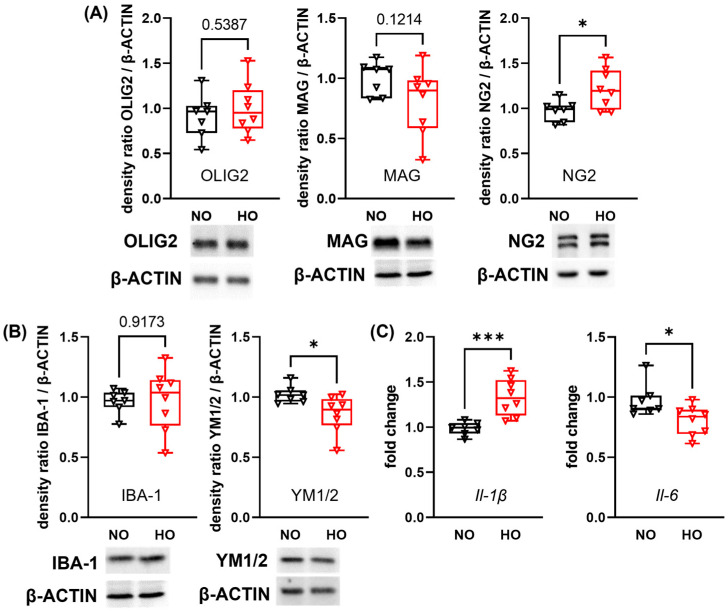
Postnatal hyperoxia leads to persistent alterations in protein and gene expression of markers for oligodendrocyte maturation and neuroinflammation. After 14 days of hyperoxia or normoxia exposure from P0 to P14 followed by two weeks in room-air, brains of 28 days-old mice were removed. Target protein was normalised to β-ACTIN and exemplary protein bands are shown below each western blot analysis graph. Oligodendrocyte maturation was investigated via western blot analysis for the pan-oligodendrocyte marker OLIG2, myelin-associated glycoprotein (MAG) for mature oligodendrocytes, and the immature oligodendrocyte marker neural glial-antigen 2 (NG2) (**A**). The protein expression of ionised calcium-binding adapter molecule-1 (IBA-1) and rodent-specific chitinase-like protein 1 and 2 (YM1/2) was analysed to evaluate microglia responses (**B**). Genes associated with microglia polarisation were analysed with real-time PCR for the pro-inflammatory cytokines interleukin-1β (*Il-1β*) and *Il-6* (**C**). Data are presented as box plots with individual data points including median values, as well as the 25% and the 75% percentile. NO: *n* = 7 animals; HO: *n* = 8 animals; * *p* < 0.05, *** *p* < 0.001.

Since neuronal and glial maturation are closely interrelated, we investigated the impact of hyperoxia on protein expression of markers of oligodendrocyte maturation. The expression levels of the pan-oligodendrocyte marker OLIG2 and the myelin-associated glycoprotein (MAG), a marker for mature oligodendrocytes, were not affected at P28 in hyperoxia-treated pups (Figure 2A). However, an increased protein expression of the immature oligodendrocyte marker neural glial-antigen 2 (NG2) was observed in the hyperoxia group (Figure 2A). Hyperoxia has been shown to induce an inflammatory response due to oxidative stress [20]. To determine whether a neuroinflammatory response is still detectable two weeks after hyperoxia exposure, we analysed the pan-microglia marker ionised calcium-binding adapter molecule-1 (IBA-1; Figure 2B). Although the amount of IBA-1 expression did not differ, the anti-inflammatory (M2) marker rodent-specific chitinase-like protein 1 and 2 (YM1/2) was decreased in hyperoxia-exposed animals (Figure 2B). Additionally, we analysed a broad spectrum of inflammatory genes associated with microglial cell polarisation, demonstrating a long-lasting and pronounced effect on mRNA expression levels of M1 markers such as elevated interleukin 1β (*Il-1β)*, diminished *Il-6* (Figure 2C), as well as slight increase of nitric oxide synthase-2 (*Inos*) and the M2 marker arginase-1 (*Arg-1*) (Figure S4). Additional M1 markers, i.e., *Cd86*, *Il-18*, tumor necrosis factor-α (*Tnf-α*) and the M2 marker transforming growth factor-β (*Tgf-β*) were not altered at P28 (Figure S4).

## 4. Discussion

Given the close association between BPD and adverse neurodevelopmental outcome, analysis of neonatal brain injury in hyperoxia-based BPD models is of critical importance [5]. Here we demonstrated that, despite recovery in room air for two weeks after hyperoxia (P0 to P14), a long-lasting neuroinflammatory response was detected. In support of this, Buczynski and colleagues showed a long-term activation of microglial cells in 8–10 months-old mice after neonatal hyperoxia from P0 to P4 with 100% oxygen [21]. While we did not detect an increase in IBA-1, we observed a shift in the expression of microglia polarisation markers with a downregulation of the M2 marker YM1/2 and an upregulation of the pro-inflammatory cytokine *Il-1β*. Previous *in vitro* experiments support that microglia react with a pro-inflammatory response to hyperoxia through an elevation of pro-inflammatory M1 molecules like IL-1β and iNOS [22]. Similar to our study, it was further shown that IL-1β expression remained elevated in the corpus callosum of rats following two weeks of mechanical ventilation [23]. The immunomodulatory cytokine IL-6 has a double-edged role not only after injury but also in development, participating in the modulation of inflammatory processes and neuronal development [24]. *Il-6* mRNA levels were upregulated immediately after 14 days of hyperoxia exposure [25]. Interestingly, we observed a downregulation of *Il-6* two weeks after the end of hyperoxia, indicating compensatory mechanisms that may be related to the impaired neurodevelopment observed in this study. Nevertheless, further experiments using isolated microglia cells are needed to assess the long-term impact of neonatal hyperoxia on this cell type and its polarisation at P28.

The sustained neuroinflammatory response could be explained by ongoing dysregulated signalling pathways in the brain. Hyperoxia induces increased cytokine expression in the brain, leading to neuronal cell death, impaired neuronal connectivity, and plasticity in the neonatal brain [2]. Interestingly, gene ontology analysis in the subventricular zone and hippocampal tissue of 12-month-old mice exposed to neonatal hyperoxia for 14 days, demonstrated long-term transcriptional modulations in neurotransmitter signalling, neuronal differentiation and vascular autoregulation important for brain development. Additionally, increased oxidative stress was still detectable in the subventricular zone of 12-month-old mice [11], which may be associated with a neuroinflammatory response. Another hypothesis might be that inflammatory molecules from the periphery penetrate the blood-brain barrier (BBB) and induce or increase neuroinflammatory processes. We detected an increased endothelial activation, presented by elevated *Icam-1* expression levels, which may lead to an enhanced interaction with peripheral leukocytes, likely leading to increased permeability and vulnerability of the BBB [26]. Additionally, *Nrp-1* and *Vegf-a*, important factors of angiogenesis and vascular remodelling, were upregulated in brains of hyperoxia-exposed animals. Enhanced levels of *Nrp-1* and *Vegf-a* were recently associated with increased growth of immature and leaky vessels [27,28]. A more permeable BBB and leaky vessels may facilitate the extravasation of pro-inflammatory cytokines or extracellular vesicles. The latter was elegantly shown for extracellular vesicles from blood samples of hyperoxia-exposed or ventilated rat pups, which led to an elevated activation of microglia and an increased expression of pro-inflammatory cytokines after intravenous injection into naïve neonatal rats [23,29]. Nevertheless, further analyses are required to identify the cause of the sustained neuroinflammation. Another aspect to consider is that the upregulation of *Nrp-1* and *Vegf-a* may represent a counteracting response to the rarefication of the vascular architecture in brains of hyperoxia-exposed animals [11]. Therefore, immunohistochemistry analysis of endothelial cell proliferation and 3D analysis of developmental vascularisation are needed for a better understanding of long-lasting effects on the cerebral vasculature.

Interactions between the vasculature and neural cells are crucial for their proper nutrition and development, which is impaired by neonatal hyperoxia. For instance, hyperoxia-mediated vascular remodelling is associated with a reduction in neuronal progenitor cells and impaired neurogenesis [11]. In the present work, we provide evidence that these deficits persist until juvenile age. We detected a reduction in *Dcx* expression, while *Nestin* expression was increased, suggesting a higher proportion of neural, not yet differentiated stem cells, compared to lineage-committed neuronal precursors cells. However, we did not find differences in the expression of the postmitotic neuronal marker NeuN. Further analysis of neuronal distribution on a cellular level would validate potential changes of cell densities in specific cortical layers. Similarly, proliferation of neural precursor cells in the neurogenic niches of the subventricular and subgranular zone need to be assessed in future studies.

Besides impaired neuronal development, we detected a lasting reduction in NG2, a marker for immature oligodendrocytes, whereas expression of the pan-marker OLIG2 and MAG, a marker for mature oligodendrocytes, were not altered. These results indicate that while oligodendrocyte numbers and myelination seem intact at that time point of development, the physiological pool of immature oligodendrocytes, needed for finalisation of myelination until early adulthood, is reduced. This might be important for overall neurodevelopment but also to resist additional insults in later life, when remyelination is needed, e.g., in autoimmune diseases or ischemic events [30,31]. Interestingly, short hyperoxia for 48 h leads to an increased expression of NG2 in the corpus callosum after 4 days of recovery under normoxia [32]. We analysed the NG2 expression 14 days after hyperoxia, highlighting the dynamic changes of myelination processes in response to neonatal hyperoxia during neurodevelopment.

This study has strength and limitations. The study is only based on a small number of animals from two independent experimental approaches. Due to storage issues of snap-frozen brains, the morphological integrity of the hemispheres was not preserved. Therefore, immunohistochemical validation of the observed changes in gene and protein expression could not be performed. Gene and protein expression analyses were conducted on whole brain hemispheres, which precludes the identification of specific neural cell types responsible for the altered secretion of cytokines and growth factors. To address these limitations and ensure the robustness and reproducibility of our findings future studies with larger cohorts are planned. These will include improved tissue preservation to enable immunohistochemistry analysis, as well as isolated local immune cells, i.e., microglia from the brain, enabling evaluation of hyperoxia-induced changes on a cellular level. However, we detected long-lasting changes in the expression of markers associated with neuronal and oligodendrocyte maturation, accompanied by persistent alterations of neuroinflammatory and vascular responses at juvenile age in response to 14 days of hyperoxia at the beginning of life. Given that children with BPD are predisposed to neurological deficits, we provide new molecular insights to hyperoxia-mediated brain injury in context to BPD, emphasising the need for in-depth investigation of adverse neurodevelopmental effects in this combined disease setting. These include, for example, neurobehavioural tests, as previous studies in EoP and BPD models showed neurodevelopmental impairments. For instance, a water maze test in juvenile rats demonstrated a reduced memory function, indicated by a longer latency time and distance to reach the platform in animals treated with hyperoxia for 14 days [13]. In addition, we demonstrated impaired spatiotemporal learning in adolescent and adult rats after 24 h of hyperoxia initiated at P6 [7]. A further key question to be answered in future studies will be whether brain and lung injury arise independently as a result of excessive oxygen exposure or whether the two organs influence each other.

## Data Availability

Original data shown in this manuscript are available from the corresponding author upon reasonable request.

## Supplementary Materials

The following supporting information can be downloaded at https://www.mdpi.com/article/10.3390/cells14151141/s1. Figure S1: Full-length western blots; Figure S2: Delta CT values of gene expression analysis; Figure S3: Growth factors and synaptic molecules important for a proper neuronal development and synaptic signal transmission were not modulated by perinatal hyperoxia; T: Hyperoxia did not alter gene expression of M1 and M2 microglia polarisation marker; Table S1: Antibodies used for western blot analysis; Table S2: TaqMan Assays used for mRNA expression analysis.

## Abbreviations

The following abbreviations are used in this manuscript:

ARG-1	Arginase-1
BBB	Blood-brain barrier
BPD	Bronchopulmonary dysplasia
CALB	Calbindin-1
CD86	Cluster of differentiation 86
DCX	Doublecortin
EGF	Endodermal growth factor
EoP	Encephalopathy of prematurity
FGF2	Fibroblast growth factor-2
HO	Hyperoxia
IBA-1	Ionised calcium-binding adapter molecule-1
ICAM-1	Intracellular adhesion molecule-1
IGF	Insulin-growth factor-1
IHC	Immunohistochemistry
IL-18	Interleukin-18
IL-1β	Interleukin-1β
IL-6	Interleukin-6
INOS	Nitric oxide synthase-2
M1	Pro-inflammatory microglia marker
M2	Anti-inflammatory microglia marker
MAG	Myelin-associated glycoprotein
NeuN	Postmitotic neuronal marker
NG2	Neural glial-antigen-2
NO	Normoxia
NRG-1	Neuregulin-1
NRP-1	Neuropilin-1
OLIG2	Oligodendrocyte transcription factor
P	Postnatal day
SYN	Synaptic molecules like synaptophysin
TBS	Tris-buffered saline
TBS-T	Tween 20 in Tris-buffered saline
TGF-β	Transforming growth factor-β
TNF-α	Tumor necrosis factor-α
VEGF-a	Vascular endothelial growth factor-a
YM1/2	Rodent-specific chitinase-like protein 1 and 2
