# Supplementary material for: Prolonged Exposure to Neonatal Hyperoxia Impairs Neuronal and Oligodendrocyte Maturation Associated with Long-Lasting Neuroinflammatory Responses in Juvenile Mice
Source: Cells. 2025 Jul 24;14(15):1141. doi: 10.3390/cells14151141 (PMC12346826; doi:10.3390/cells14151141)
Supplement: Supplementary file 1 [file cells-14-01141-s001.zip › cells-3721945-supplementary.pdf]

Supplement

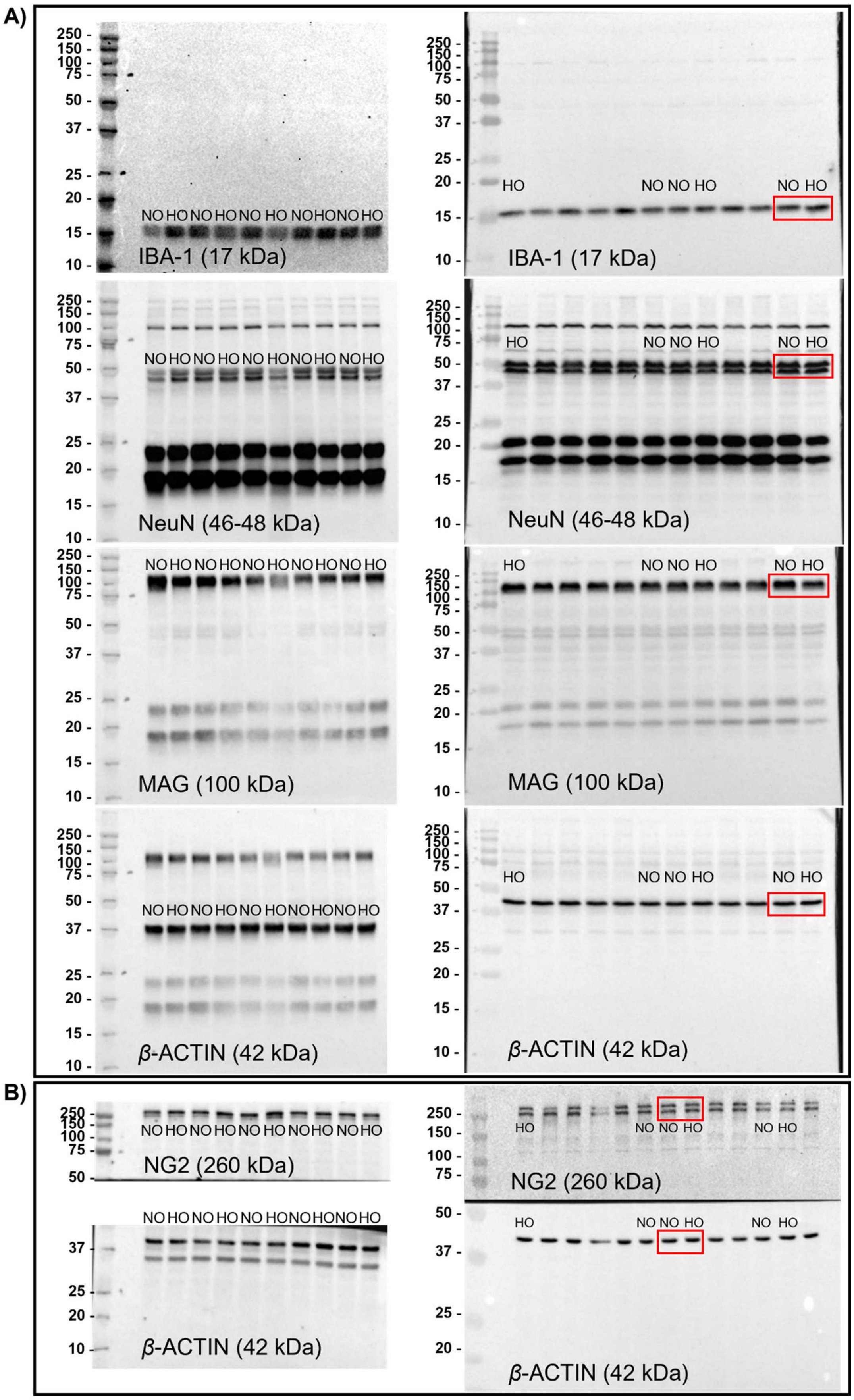

See the next page for continued figure.

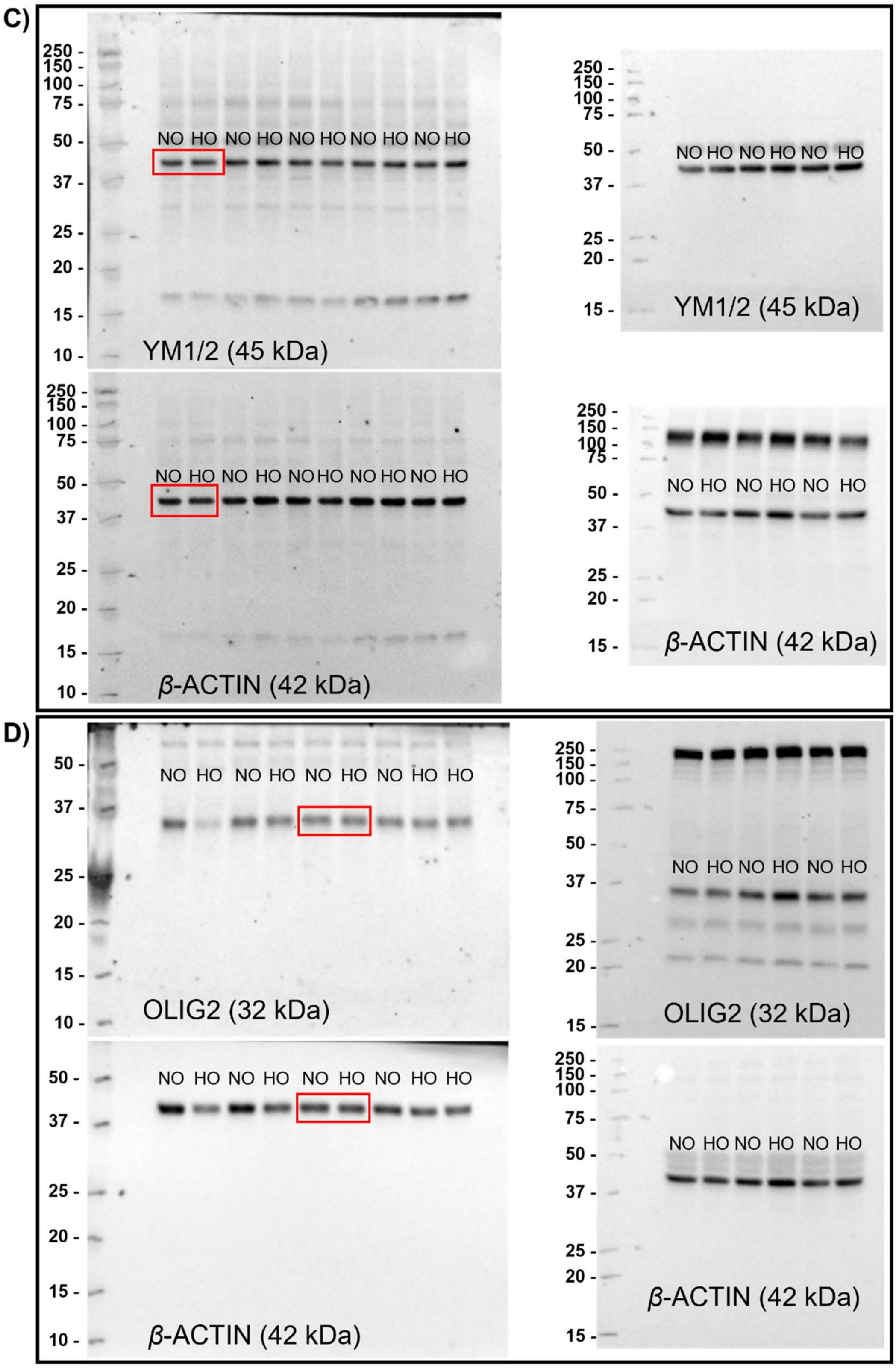

Figure S1: Full-length western blots. All images represent merged images of original western blot images and transmitted light images to visualise ladder bands (marker). IBA-1, NeuN, MAG and β-ACTIN (A) were analysed on the same membrane. To avoid cross reactions of primary or secondary antibodies membranes were cut at approximately 50 kDa prior to antibody incubation and detection. Upper membrane was used for NG2 and the lower membrane was used for β-ACTIN detection (B). Animals from two independent experiments were used for analysis. One batch was randomly loaded with samples of another project (A and B: right membranes, lanes without labels), which are not part of this study. YM1/2 (C) and OLIG2 (D) were analysed on different membranes. Each target protein was

normalised to β-ACTIN detected on the same membrane. Cropped regions of target proteins used for representation in the main figure are indicated by red boxes.

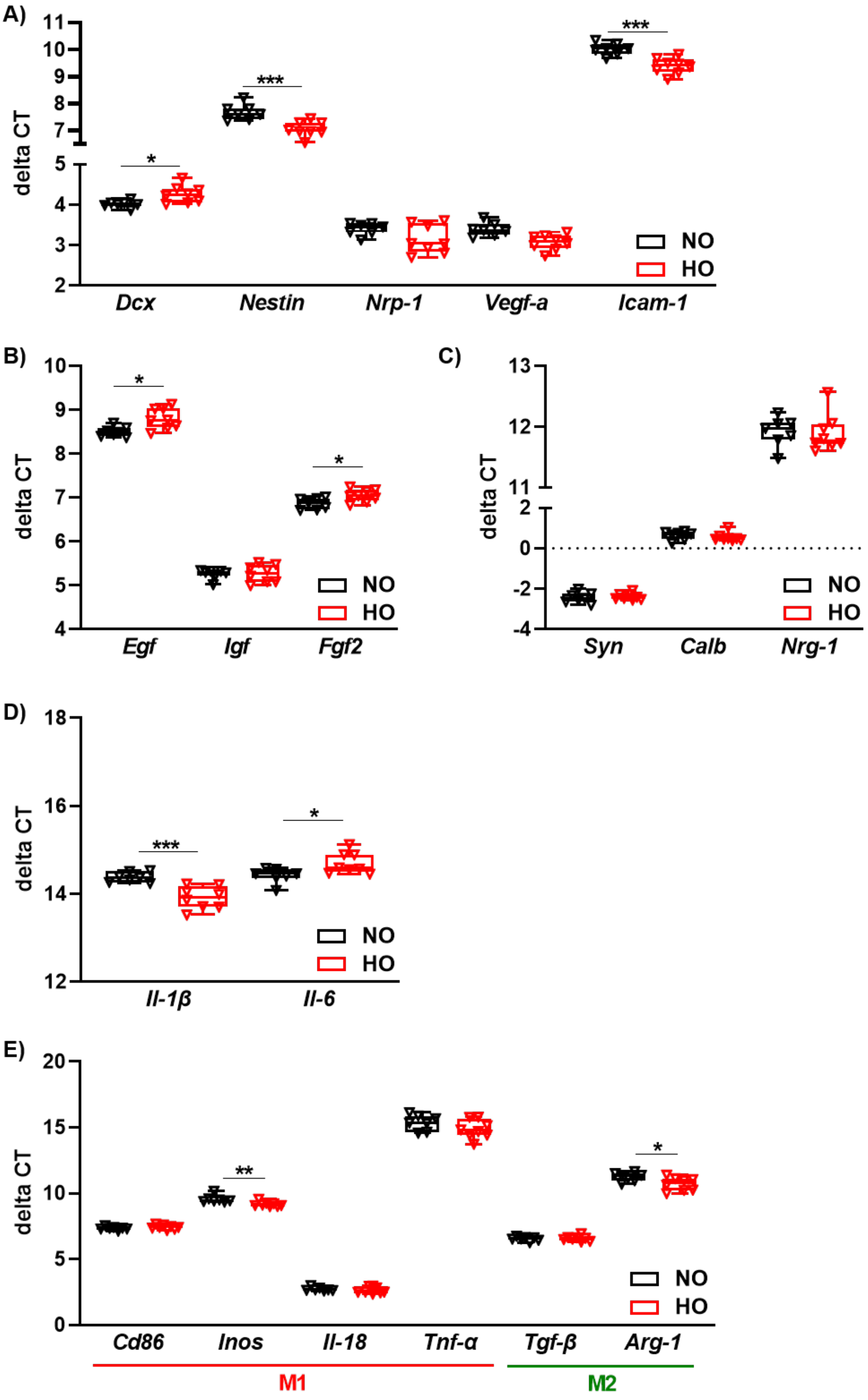

Figure S2: Delta CT values of gene expression analysis. Real time PCR was performed of brain lysates of the right hemisphere. Delta CT values are plotted for genes *Dcx and Nestin, associated with neuronal maturation; Nrp-1*, *Vegf-a* and *Icam-1, as vascularisation markers* (A); growth factors *Egf*, *Igf* and *Fgf2 (B);* synaptic molecules *Syn*, *Calb*, and *Nrg-1* (C); cytokines *Il-1β and Il-6* (D); pro-inflammatory M1 markers *Cd86*, *Inos*, *Il-18*, and *Tnf-α* as well as anti-inflammatory M2 markers *Tgf-β* and *Arg-1* (E). Data are

presented as box plots with individual data points including median values, the 25% and the 75% percentile; NO: n = 7 animals; HO: n = 8 animals; *$p < 0.05$, **$p < 0.01$, ***$p < 0.001$.

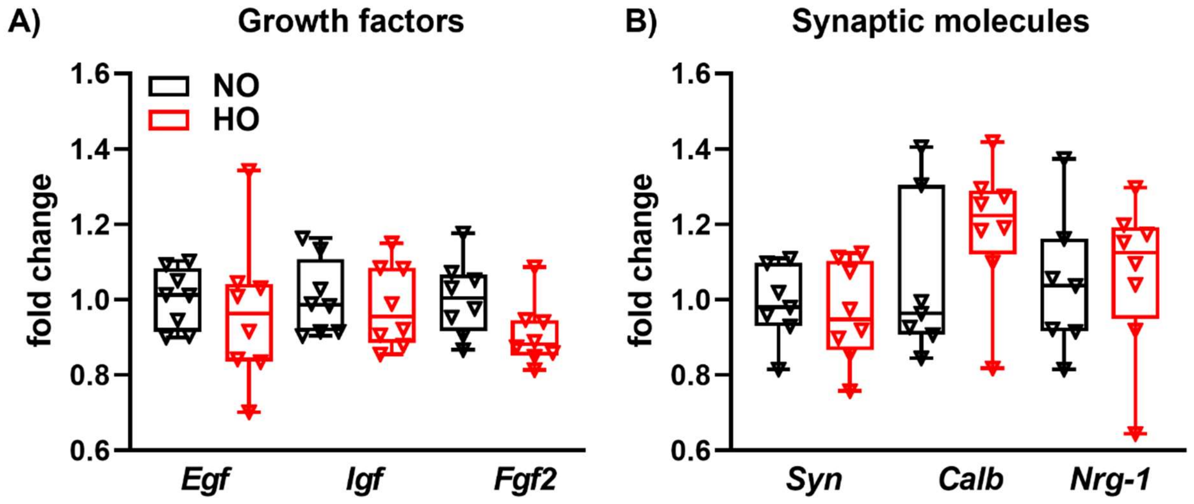

Figure S3: Growth factors and synaptic molecules important for a proper neuronal development and synaptic signal transmission were not modulated by perinatal hyperoxia
After 14 days of hyperoxia (HO) or normoxia (NO) and additional 14 days of normoxia, brains were removed at P28. Gene expression analysis was performed for epidermal growth factor (*Egf*), insulin growth factor (*Igf*) and fibroblast growth factor (*Fgf2*) (A) as well as for synaptic molecules such as synaptophysin (*Syn*), calbindin-1 (*Calb*) and neuregulin-1 (*Nrg-1*) (B) from brain lysates of the right hemisphere. NO: $n = 7$ animals; HO: $n = 8$ animals.

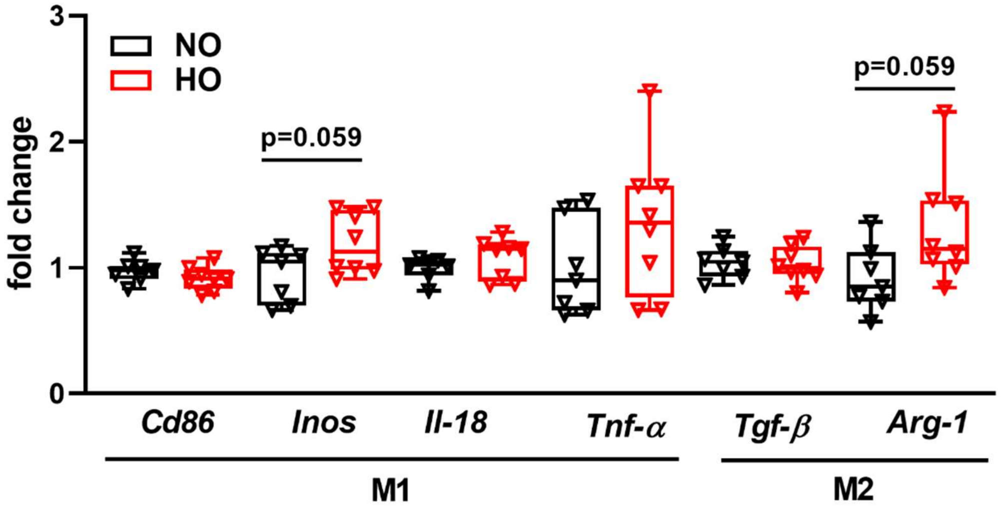

Figure S4: Hyperoxia did not alter gene expression of M1 and M2 microglia polarisation marker
Brains were removed at P28 after two weeks of hyperoxia (HO) or normoxia (NO) from P0 - P14. Gene expression analysis was performed for microglia M1 marker *Cd86*, nitric oxide synthase-2 (*Inos*), interleukin-18 (*Il-18*), and tumor necrosis factor-α (*Tnf-α*) as well as M2 markers transforming growth factor-β (*Tgf-β*) and arginase-1 (*Arg-1*) in brain lysates of the right hemisphere. NO: $n = 7$ animals; HO: $n = 8$ animals.

Table S1: Antibodies used for western blot analysis

Antigen	blocking	dilution	supplier	sec. antibody (supplier)	dilution (sec. antibody)
**IBA-1**	5% MP/TBS-T	1:3000	Wako	anti-rabbit (Cell Signaling)	1:2000
**MAG**	5% MP/TBS-T	1:2000	Abcam	anti-mouse IgG 1 (Novus Biologicals)	1:5000
**NeuN**	5% MP/TBS-T	1:2000	Millipore	anti-rabbit (Cell Signaling)	1:2000
**NG2**	5% BSA/TBS-T	1:1000	Cell Signaling	anti-rabbit (Cell Signaling)	1:2000
**OLIG2**	5% MP/TBS-T	1:2000	Millipore	anti-rabbit (DAKO)	1:2000
**YM1/2**	5% MB/TBS-T	1:3000	Abcam	anti-rabbit IgG (Novus Biologicals)	1:5000
**β-ACTIN**	5% MB/TBS-T	1:20000	Sigma	anti-mouse (DAKO)	1:5000

Abbreviations: BSA = bovine serum albumin, MP = non-fat milk powder, TBS-T = 0.1% Tween20 in Tris-buffered saline

Table S2: TaqMan Assays used for mRNA expression analysis

Gene	Abbreviation	Assay ID
Arginase-1	*Arg-1*	Mm00475988_m1
β-2-microglobulin	*B2m*	Mn00437762_m1
Calbindin-1	*Calb*	Mm00486647_m1
Cd86	*Cd86*	Mm00444543_m1
Doublecortin	*Dcx*	Mm00438400_m1
Epidermal growth factor	*Egf*	Mm00438696_m1
Fibroblast growth factor-2	*Fgf2*	Mm01285715_m1
Intracellular adhesion molecule-1	*Icam-1*	Mm00516023_m1
Interleukin-18	*Il-18*	Mm00434226_m1
Interleukin-1β	*Il-1β*	Mm00434228_m1
Interleukin-6	*Il-6*	Mm00446190_m1
Insulin-like growth factor-1	*Igf*	Mm00439560_m1
Nestin	-	Mm00450205_m1
Neuropilin-1	*Nrp-1*	Mm00545877_m1
Nitric oxide synthase-2	*Inos*	Mm00440502_m1
Sox10	*Sox10*	Mm00569909_m1
Synaptophysin	*Syn*	Mm00436850_m1
Transforming growth factor-β	*Tgf-β*	Mm01178820_m1
Tumor necrosis factor-α	*Tna-α*	Mm00443258_m1
Vascular endothelial growth factor-a	*Vegf-a*	Mm00437306_m1
